# Improving Medication Regimen Recommendation for Parkinson’s Disease Using Sensor Technology

**DOI:** 10.3390/s21103553

**Published:** 2021-05-20

**Authors:** Jeremy Watts, Anahita Khojandi, Rama Vasudevan, Fatta B. Nahab, Ritesh A. Ramdhani

**Affiliations:** 1 Department of Industrial and Systems Engineering, University of Tennessee, Knoxville, TN 37996, USA; gkm819@vols.utk.edu (J.W.); khojandi@utk.edu (A.K.); 2 Center for Nanophase Materials Science, Oak Ridge National Laboratory, Oak Ridge, TN 37830, USA; vasudevanrk@ornl.gov; 3 Department of Neurosciences, University of California San Diego, La Jolla, CA 92093, USA; fnahab@ucsd.edu; 4 Department of Neurology, Donald and Barbara School of Medicine at Hofstra/Northwell, Hempstead, NY 11549, USA

**Keywords:** Parkinson’s disease, wearable sensors, machine learning, levodopa, regimen, decision support tool, remote assessment, PKG, clustering

## Abstract

Parkinson’s disease medication treatment planning is generally based on subjective data obtained through clinical, physician-patient interactions. The Personal KinetiGraph™ (PKG) and similar wearable sensors have shown promise in enabling objective, continuous remote health monitoring for Parkinson’s patients. In this proof-of-concept study, we propose to use objective sensor data from the PKG and apply machine learning to cluster patients based on levodopa regimens and response. The resulting clusters are then used to enhance treatment planning by providing improved initial treatment estimates to supplement a physician’s initial assessment. We apply k-means clustering to a dataset of within-subject Parkinson’s medication changes—clinically assessed by the MDS-Unified Parkinson’s Disease Rating Scale-III (MDS-UPDRS-III) and the PKG sensor for movement staging. A random forest classification model was then used to predict patients’ cluster allocation based on their respective demographic information, MDS-UPDRS-III scores, and PKG time-series data. Clinically relevant clusters were partitioned by levodopa dose, medication administration frequency, and total levodopa equivalent daily dose—with the PKG providing similar symptomatic assessments to physician MDS-UPDRS-III scores. A random forest classifier trained on demographic information, MDS-UPDRS-III scores, and PKG time-series data was able to accurately classify subjects of the two most demographically similar clusters with an accuracy of 86.9%, an F1 score of 90.7%, and an AUC of 0.871. A model that relied solely on demographic information and PKG time-series data provided the next best performance with an accuracy of 83.8%, an F1 score of 88.5%, and an AUC of 0.831, hence further enabling fully remote assessments. These computational methods demonstrate the feasibility of using sensor-based data to cluster patients based on their medication responses with further potential to assist with medication recommendations.

## 1. Introduction

Parkinson’s disease (PD) is a neurodegenerative disorder resulting from the loss of dopaminergic neurons. It is characterized by four cardinal motor symptoms: Bradykinesia (slowing of movement), muscle rigidity, tremor, and postural instability/gait disorder. Additionally, symptoms, such as rapid eye movement sleep behavioral disorder (RBD), anosmia, and constipation, can present as prodromes, while other nonmotor symptoms—bladder dysfunction, dysphagia, orthostatic hypotension, and cognitive impairment—can manifest later in the disease course [1]. The number of individuals diagnosed with PD is estimated to be 6.2 million globally [2], and approximately 60,000 individuals are diagnosed with PD annually in the U.S. alone [3]. Studies have reported demographic differences with respect to sex and race in PD diagnosis frequency [4,5,6]. Individuals living with PD are expected to increase as global life expectancy increases; this will place additional strain on the medical system. Due to the shortage of neurologists and logistical challenges, including extended travel time, patient disability, and prolonged clinic wait times, PD patients tend to have fewer clinic visits [7,8]. Women, racial minorities, and rural communities have less access to care and lower quality of specialist care [7], causing delays in diagnosis and higher long-term disability [9,10]. It is further expected with the increasing number of PD patients, these inequities to the quality of care will become more prevalent [3]. Currently, these clinic visits are critical to improving an individual’s treatment planning and represent a potential bottleneck in the quality of patients’ care.

Levodopa remains the gold standard therapy for treating the cardinal motor symptoms of PD. As Parkinson’s progresses, the duration of levodopa’s dose efficacy shortens with the emergence of motor complications, such as: “Wearing-off” episodes (a return of motor symptoms prior to taking the next dose); “delayed ON periods” (slow onset of dose benefit); “On-Off cycling” or “motor fluctuations” (symptomatic benefits are achieved during the ON phase of the dose followed by OFF periods characterized by uncontrolled motor symptoms prior to the next dose); “dyskinesia” (involuntary movements affecting the limb). These complications result from various factors, including disease progression and pulsatile stimulation of dopamine receptors, due to lack of continuous levodopa administration [11]. Typically, a patient’s medication regimen is optimized by fragmenting and increasing levodopa dosages, while utilizing monoamine oxidase B (MAO-B), dopamine agonists, or catechol-O-methyl transferase (COMT) inhibitors as adjunctive therapies to provide dopamine replacement. The primary goal for PD treatment is to optimize symptom control, while minimizing off periods and medication side effects. 

Currently, assessments of the efficacy of patients’ treatments are based on a clinician’s overall impression of motor disability as determined by clinical assessment tools, such as the MDS-Unified Parkinson’s disease Rating Scale (UPDRS) [12] and Hauser paper-based diaries [13]. The lack of continuous motor assessment coupled with recall bias and limited integration of nonmotor symptomatology into the treatment paradigm present real-world limitations in managing such a heterogeneous condition. Sensor-based technology offers a real-time mechanism to objectively measure motor performance in PD [14,15], moving beyond the “snapshot” clinical assessment of impairment. 

**Figure 1:**
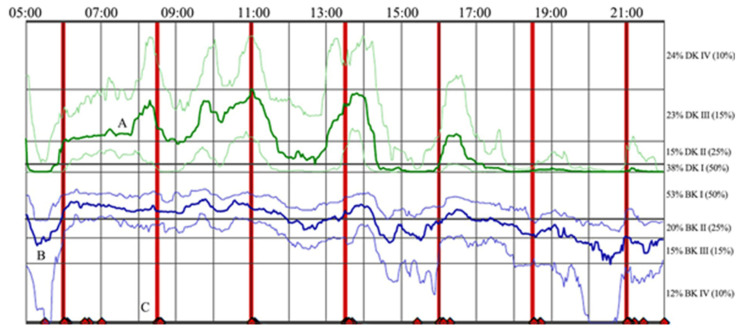
Sample PKG sensor time-series data [23], displaying an individual’s change in dyskinesia and bradykinesia scores in response to medication with the median, 25th, and 75th percentile, compared to a non-PD control group averaged over six days. (**A**) The dyskinesia time-series, (**B**) the bradykinesia time-series, and (**C**) the patient’s self-reported acknowledgment of medication administration.

Specialty PD motor sensors have demonstrated 70–90% accuracy in measuring fluctuations and dyskinesia in patients’ medication response [16,17,18,19]. One such inertial sensor is the Personal KinetiGraph™ (PKG) sensor (Global Kinetics Corporation (GKC), Melbourne, Australia). This wrist-worn logger utilizes an accelerometer to collect movement information in two-minute intervals and reminds patients to register when taking their prescribed dopaminergic medication. The raw data are converted into summary dyskinesia and bradykinesia scores (averaged single value assessments over the entire wear period), as well as time-series data, curated into a report [18] using validated algorithms [18,20,21,22]. The report shows the continuous changes of dyskinesia and bradykinesia scores, as it relates to levodopa timing as the median, 25th, and 75th percentile, compared to a non-PD control group over six days. A sample PKG report is provided in Figure 1.

By examining a spectrum of patients with clinical variability and gauging their responsivity to dopaminergic medication with sensor technology, inherent medication similarities may be present within specific clinical subtypes—offering an opportunity to cluster patients in a treatment-related manner. This approach could serve to predict optimal regimens, potentially reducing the lengthy process of optimizing medication for patients. Additionally, this approach could improve the equity in PD treatment planning by utilizing remote monitoring to reduce the need for difficult clinic visits. Strategic treatment planning using PD patient subtyping has been shown effective [24,25] and stands to offer a data-driven approach to refining clinical management. Therefore, this study is a proof-of-concept to examine the feasibility of determining clinically relevant patient regimen clusters and identifying these clusters based on symptoms measured by wearable sensors to be utilized in the determination of future patients’ treatment plans.

## 2. Materials and Methods

### 2.1. Study Cohort

Characteristics of the patient cohort and selection process are thoroughly described in the study by Nahab et al. [23], which explored the clinical utility of the PKG in the routine care of Parkinson’s patients. All patients were selected from the UCSD Movement Disorder Center from June 2016 to March 2017. The study’s inclusion criteria included: An age range of 46–83, being on levodopa, and Hoehn and Yahr stages 1–3 [23]. Patients were excluded if they had been previously diagnosed with dementia that could impact their use of the wearable sensor [23]. The participants underwent two clinical assessment visits. Before each visit, the PKG sensor was worn by the patient for a six-day period, over which patients’ key symptoms, namely, dyskinesia and bradykinesia, were scored every two minutes throughout the patient’s full monitoring day (approximately 17 h). During the clinic visits, the physician assessed MDS-UPDRS motor subscales III & IV [12] were conducted. After the first study visit, a management plan, including an updated medication regimen, was developed based on the PKG report and clinical assessments [23]. The patient then followed the updated management plan, while monitored by the PKG sensor for another six-day period. The patients were then evaluated in the second visit by the same clinical metrics, including the PKG. The change in patient’s symptom control based on the updated management plan was determined. In this study, we retrospectively evaluate patients’ symptom control under both management plans in the cohort assessed by Nahab et al. [23].

### 2.2. Study Design

**Figure 2:**
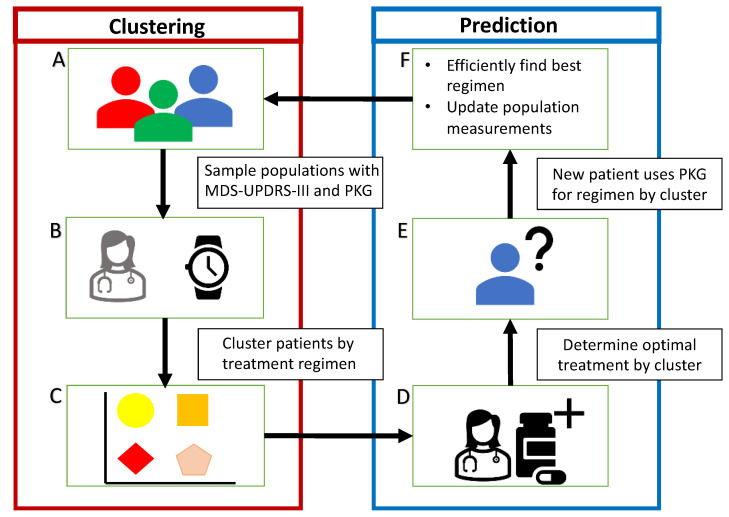
The study design is divided into two parts as labeled by the blocks “Clustering” and “Prediction”. In the clustering block, (**A**) we begin with a diverse cohort of PD patients; (**B**) each patient is assessed by MDS-UPDRS-III scores and PKG summary dyskinesia and bradykinesia scores; the physician icon is greyed out as in the future for some remote contexts this may be accomplished using only the PKG time-series data, but we currently collect both for validation purposes; (**C**) we identify similar medication regimen clusters through k-means clustering. These clusters are used in the prediction block; (**D**) we optimize medication regimens and perform statistical analysis on demographic similarities for each group—to create a decision support tool to provide enhanced initial regimen estimates; (**E**) machine learning methods, specifically random forest, are applied to predict an unknown patient’s optimized regimen cluster based on physician assessment and/or wearable sensor measurements depending on the context; (**F**) the new patient’s data will be incorporated to improve the accuracy of the decision support tool.

We seek to group patients between clusters based on their optimized clinical medication regimens to determine if clinically relevant clusters exist within the patient cohort. Such patient clusters are thought to exist within cohorts, but may not be identifiable by demographic information alone. To this end, we also examine the role of MDS-UDPRS-III scores and PKG time-series data to identify these clusters. These clusters would allow for the rapid estimation of near-optimal medication regimens for new patients. By examining within-subject symptom change during the optimization process of patients’ medication regimens, we are seeking the patients’ “best” performing medication regimens. The cluster allocation of new patients could then be predicted, placing the new patients within clusters that, on average, perform best (i.e., minimizes patients’ symptoms based on cohort level estimations). While an individual’s PD symptoms are unique, such an average best performing regimen could provide a clinician with an improved starting point for treatment planning, reducing the need for lengthy clinical assessments. The study design is shown in Figure 2. 

We apply a statistical clustering technique to group patients based on their medication regimens under various conditions. Specifically, we examine their visit two regimens (during the physician-led optimization process) and their best performing medication regimen (being visit 1 or visit 2 when their symptoms were best controlled). These clusters and regimens are then compared to identify significant features which may aid in their consistent prediction.

We perform a comparative analysis between patients’ MDS-UPDRS-III scores and PKG’s summary dyskinesia and bradykinesia scores during treatment optimization to determine the efficacy of using wearable sensors for symptom management. To accomplish this, we examine the within-subject symptom change under both visits’ regimens and determine which visit regimen best controlled a patient’s symptoms as assessed by both MDS-UPDRS-III scores and the PKG’s summary dyskinesia and bradykinesia scores. We compare these optimized regimens for each patient to examine discrepancies in patients’ symptom assessments between the MDS-UPDRS-III and the PKG scores. We then examine patients’ demographic information (study age, age at diagnosis, years of PD, and gender) under each clustering condition to identify statistically significant differences between similarly optimized regimens to determine if demographic information alone may uniquely identify an optimized cluster.

Following the identification of optimized patient regimens based on the best clustering scheme, we apply machine learning techniques to predict the optimal medication regimens of patients through a combination of features. We examine the role of demographic information, MDS-UPDRS-III scores, and PKG time-series data in predicting the cluster allocation of patients. Such a prediction would create a decision support tool that could estimate a patient’s optimized regimen aiding physicians. Further, we examine the potential of predictive algorithms without using traditional clinical symptom assessment methods (MDS-UPDRS-III) instead of based solely on wearable sensor measurements. We provide a machine learning algorithm with the patients’ visit 1 PKG time-series data and predict their generalized optimal regimen. Being able to predict accurate estimates of a patient’s optimal regimen remotely would save clinical time, equalize healthcare opportunities, and place less burden on patients during the process of medication optimization. 

### 2.3. K-means Clustering

K-mean clustering is an unsupervised machine learning algorithm that partitions patients into a predetermined number of clusters (k) without a hierarchical structure [26]. In this algorithm, clusters are initially formed, and each patient is grouped into their nearest cluster (with respect to Euclidian distance to cluster centroid). The clusters’ centroids are then recalculated, seeking to minimize the distance between patients and their assigned centroid. Patients are then reassigned to the nearest clusters. This process is performed iteratively and continues until no patients are reassigned in an update [26]. 

**Figure A1:**
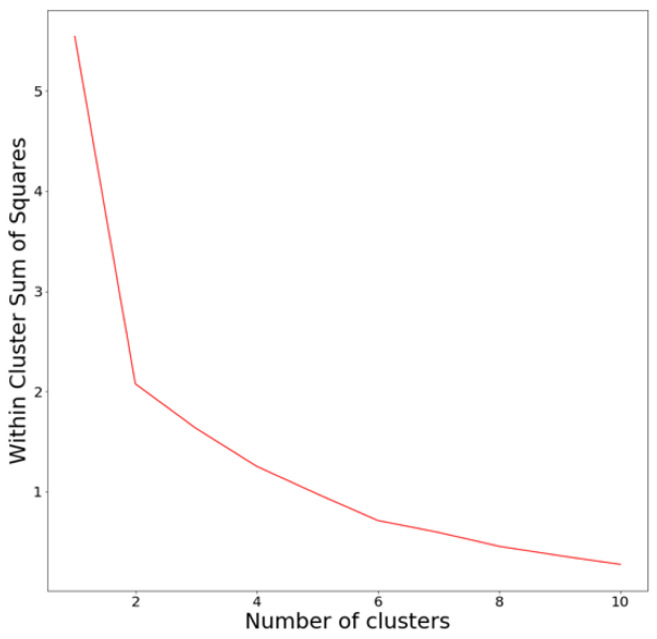
WCSS as a function of the number of clusters when clustering patients based on their best symptom control medication regimens between the two visits according to PKG’s summary dyskinesia and bradykinesia scores. The number of clusters is set to four, as a number towards the bottom of the “elbow” is considered best, but further increasing the number of clusters has been shown to reduce the model’s robustness.

Daily total levodopa equivalent dose (calculated by converting each PD drug to levodopa equivalent doses (LED) and cumulating them), daily total carbidopa/levodopa IR (immediate release) dose, which is the common dopamine replacement agent utilized in PD drug regimens, and levodopa administration frequency were used in k-means clustering. These regimen features were used as each is likely to be modified in the physician-led optimization process. The number of clusters (k) was determined per the Within Cluster Sum of Squares (WCSS) measurement, which minimizes the within-cluster variance (e.g., the results of this analysis are provided in Figure A1 in Appendix A). This technique has been effectively used in healthcare applications for clustering data [27]. The WCSS resulted to identify four clusters to meaningfully separate the patient cohort. Consequently, we evaluated demographic information (patient’s age at visit 1, age at diagnosis, number of years experiencing PD symptoms, and gender) for each cluster under three clustering schemes. 

### 2.4. Random Forest

A random forest classifier is a supervised machine learning algorithm that utilizes a large number of decision trees working as an ensemble [28]. We opt to use the random forest classifier in this study as it is generally very robust against noisy or high dimensional datasets; it is not susceptible to overfitting [29]. Four random forest classifiers were trained to stratify subjects into their designated clusters—identified based on the patients’ best medication regimen according to PKG’s summary dyskinesia and bradykinesia scores—using combinations of demographic information, visit 1 MDS-UPDRS-III scores, and visit 1 PKG time-series data. The PKG time-series data (representing two-minute increment measurements of the dyskinesia and bradykinesia scores) were extracted from the PKG report. Features were extracted and engineered from the PKG time-series via TSFresh [30], which calculates various time-series characteristics frequently used in classification tasks. Features importance ranking was conducted using the Gini index [31]. The topmost important features were identified prior to cross-validation during preliminary experiments. These features are provided to the reader in Table A1 in Appendix A. 

Each random forest model’s performance was evaluated using leave-one-out cross-validation. In each set, a single patient was left out of the training data on which the random forest learned then that patient’s cluster allocation as determined by their best medication regimen was predicted. This process is repeated until all patients have been used for testing, retraining the random forest model each time to prevent contamination between training and testing sets. Due to the unbalanced representation of clusters inherent in the dataset, repeated downsampling was used in each model. Repeated downsampling results in a balanced dataset for use in learning such that during training, the model did not favor the more representative cluster. Specifically, we randomly sampled from the more representative cluster to create a subset equal to the number of the less represented cluster for training each decision tree. This downsampling process was repeated for each decision tree. The total number of decision trees for each of the four random forest models was determined through preliminary experiments. Specifically, 200 decision trees were used in the demographic information, and the demographic information and visit 1 PKG time-series models, whereas 500 decision trees were used in the demographic information and visit 1 MDS-UPDRS-III model, and 100 decision trees were used in the demographic information, MDS-UPDRS-III, and visit 1 PKG time-series model.

Performance metrics included sensitivity, specificity, accuracy, positive predictive value (PPV), F1 score, and the area under the receiver operating characteristic (AUC). Sensitivity is the proportion of positives that are correctly identified. Specificity is the proportion of negatives that are correctly identified. PPV is the measurement of positive and negative results that are true positives. Accuracy is the measurement of correct predictions out of all predictions. The F1 score is the harmonic average of precision and recall. The AUC is the aggregate comparison of the true positive rate and the false positive rate at different classification thresholds and provides an overall performance metric for the model. We determined confidence intervals for each metric by repeating the random forest analysis 100 times under different initial random seeds.

## 3. Results

### 3.1. Cohort Characteristics

A total of 26 subjects (17 male and 9 female) clinical evaluations and PKG reports were included from the study by Nahab et al. [23]. The PKG reports consisted of time-series data; specifically, dyskinesia and bradykinesia scores assessed every two minutes averaged over six days, along with medication administration times. The PKG time-series data were extracted from the PKG’s reports. The patient cohort utilized in this study is a subset of that presented in [23]. Two participants were excluded from the evaluation: One participant did not have corresponding PKG reports; the other had dosage inconsistencies in the recorded medication regimen. During visit 2, the overall mean MDS-UPDRS-III score was significantly reduced (visit 1: 28.9 ± 14.1, visit 2: 24.1 ± 13.5, *p*-value < 0.028 [23]). Demographic information and clinical characteristics of the participants are provided in Table 1. This retrospective study was conducted according to the guidelines of the Declaration of Helsinki and approved by the Institutional Review Board of the University of Tennessee (UTK-IRB-20-06007-XP).

### 3.2. Patient Clustering Using Medication Regimen

K-means clustering was utilized to allocate patients into one of four clusters based on their prescribed daily total levodopa equivalent dose, daily total carbidopa/levodopa IR dose, and levodopa administration frequency. In the first experiment, subjects were clustered by their visit two regimens, in which physicians had adjusted individualized regimens to optimize motor symptoms based on the clinical MDS-UPDRS-III scores and PKG report. In the second experiment, subjects were clustered according to the regimen associated with best motor function improvement (i.e., minimizes patients’ symptoms), as defined by the MDS-UPDRS-III scores or PKG’s summary dyskinesia and bradykinesia scores, respectively.

**Figure 3:**
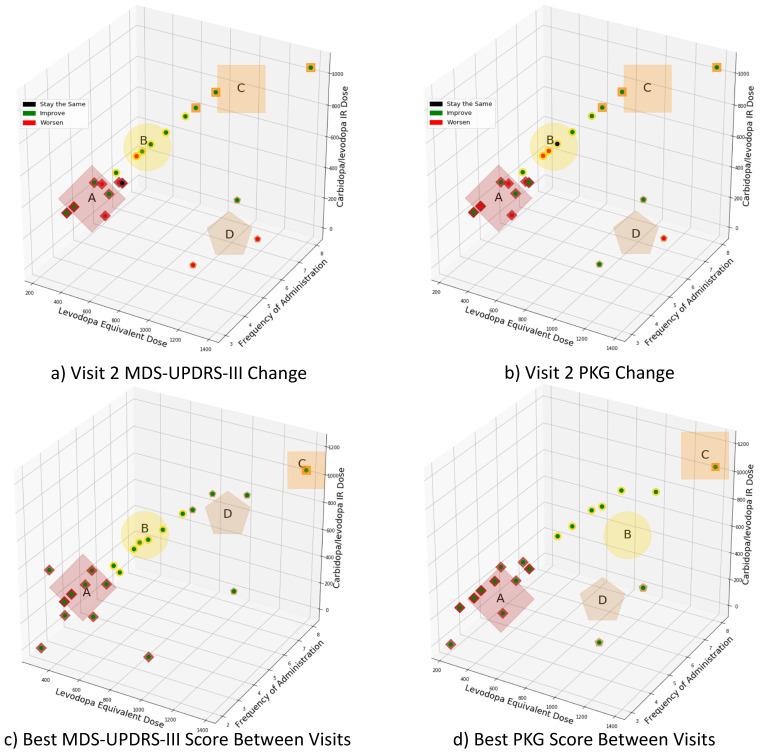
(**a**,**b**) demonstrates the clusters for the motor function changes between visit 1 to visit 2 based on the MDS-UPDRS-III scores and the PKG’s summary dyskinesia and bradykinesia scores, respectively. (**c**,**d**) highlight the subjects’ best symptom control recorded using MDS-UPDRS-III scores and PKG’s summary dyskinesia and bradykinesia scores, respectively. The large shapes denote each cluster’s centroid, and the exterior marker of each point corresponds to the cluster centroid shape. The capital letters (A–D) are used to refer to each cluster. Each point’s interior maker in (**a**,**b**) represents the state of MDS-UPDRS-III and PKG change for each patient, where a black interior-point denotes the patient stayed the same between visits, a green point denotes that the patient’s visit 2 scores were better than their visit 1 scores. A red point denotes that the patient’s visit 2 scores were worse than their visit 1 scores. Note that due to the three-dimensional projection of the plot, the distance between points may appear skewed. See Supplemental Information for a 3-D animation showcasing the clusters’ position in space.

Figure 3a,b presents the clusters based on visit 2’s medication regimen. According to Figure 3a, when MDS-UPDRS-III scores are used as the comparison metric: Eighteen subjects show symptom improvements, while seven demonstrated symptom worsening, and one remained unchanged. As shown in Figure 3b, when the PKG’s summary dyskinesia and bradykinesia scores are used: Seventeen subjects show symptom improvements, eight demonstrated symptom worsening, and one remained unchanged. The demographic information associated with each cluster is provided in Table 2. Cluster D was statistically different from clusters A and B with respect to disease duration and age at diagnosis (*p* < 0.05); no other clusters were statistically different in terms of demographic parameters (*p* > 0.05).

Figure 3c,d presents the medication regimens’ clusters associated with the best motor function between the two study visits. The centroid positions of the clusters are in different locations, since medication regimens related to improved clinical function differ according to MDS-UPDRS-III scores and PKG’s summary dyskinesia and bradykinesia scores. The patients’ demographic information for each cluster is provided in Table 2, while the breakdown of PD medication and dosing is provided in Table 3. It should be noted that for MDS-UPDRS-III scores and PKG scores that were unchanged between visit 1 and visit 2, the regimen associated with visit 2 was considered “best” and used in this clustering. No two clusters are statistically different (*p* > 0.05) in terms of gender or age at diagnosis. Cluster D was statistically different from cluster A with respect to patients’ age (*p* < 0.05) for the best MDS-UPDRS-III scores. Likewise, cluster D was statistically different from clusters A and B with respect to disease duration (*p* < 0.05) for the best PKG’s summary dyskinesia and bradykinesia scores. 

### 3.3. Random Forest Classification Using PKG Readouts

Four random forest classifiers were trained to examine the efficacy of using combinations of demographic information (patient’s age at visit 1, age at diagnosis, number of years experiencing PD symptoms, and gender), visit 1 MDS-UPDRS-III scores, and visit 1 PKG time-series data, to stratify the subjects in clusters A and B, as identified through k-means clustering using the best PKG score (see Figure 3d). As shown in Table 2, “Best PKG Score,” clusters A and B contain 17 and 6 participants, respectively. As noted in Section 3.2 (“Patient Clustering Using Medication Regimen”), clusters A and B were the most statistically similar with respect to the demographic information and contained the majority of participants. The performance of each of the classifiers is presented in Table 4.

The random forest classifier using solely demographic information achieved a sensitivity of 61.3 ± 1.0%, a specificity of 62.3 ± 1.5%, an accuracy of 61.6 ± 0.8%, a PPV of 82.2 ± 0.6%, an F1 score of 70.1 ± 0.8%, and an AUC of 0.618 ± 0.008. Whereas the random forest classifier using both demographic information and visit 1 MDS-UPDRS-III scores achieved a sensitivity of 65.2 ± 0.8%, a specificity of 66.0 ± 0.7%, an accuracy of 65.4 ± 0.6%, a PPV of 84.4 ± 0.3%, an F1 score of 73.5 ± 0.6%, and an AUC of 0.656 ± 0.005. 

The random forest classifier using demographic information and visit 1 PKG time-series data had superior performance to the subjective MDS-UPDRS-III-based classifier. To train this random forest classifier, over 1000 features were extracted from PKG sensors’ dyskinesia and bradykinesia time-series for each patient, of which the top ten most important features were included in the analysis. These features are provided in Table A1 in the Appendix A. This random forest classifier achieved a sensitivity of 84.5 ± 0.7%, a specificity of 81.7 ± 2.2%, an accuracy of 83.8 ± 0.7%, a PPV of 93.1 ± 0.8%, an F1 score of 88.5 ± 0.5%, and an AUC of 0.831 ± 0.011.

The random forest classifier using demographic information, visit 1 MDS-UPDRS-III and visit 1 PKG time-series data had the best overall performance with a sensitivity of 86.5 ± 0.5%, a specificity of 87.7 ± 1.6%, an accuracy of 86.9 ± 0.6%, a PPV of 95.3 ± 0.6%, an F1 score of 90.7 ± 0.4%, and an AUC of 0.871 ± 0.008. The PKG time-series features included in this random forest model are identical to those listed in Table A1.

## 4. Discussion

Utilizing a Parkinson’s patient cohort dataset consisting of within-subject medication regimen titrations—clinically assessed by the MDS-UPDRS-III scores and PKG’s summary dyskinesia and bradykinesia scores—k-means clustering was used to group patients in terms of daily total levodopa equivalent dose, daily total carbidopa/levodopa IR dose, and levodopa administration frequency. We demonstrate that subjects can be meaningfully clustered based on longitudinal dopaminergic treatment regimens. The sensor-based assessments of the PKG can estimate patient symptoms corresponding to similar MDS-UPDRS-III scores. Further, the PKG sensor can be thought of as enhancing the granularity of this clustering method compared with the MDS-UPDRS-III scores: When referencing cluster D, the PKG clustering has statistical significance between clusters A and B, whereas the MDS-UPDRS-III clustering only has statistical significance between cluster A.

Figure 3a,b show the difference between MDS-UPDRS-III scores and PKG’s summary dyskinesia and bradykinesia scores when determining subject improvement. This difference is quite minor between the two assessment instruments with respect to the regimens yielding the best motor function. This result supports the growing body of literature that the MDS-UPDRS-III score can be adequately determined by wearable sensor estimates [18,23,32]. This comparison shows that determining the optimization of a patient’s medication regimen may be effectively estimated using sensors. However, since the cohort was treated, considering both PKG changes and traditional clinical assessments, further conclusions regarding the robustness of the treatment approaches cannot be drawn. Therefore, our results suggest that efficiently establishing a patient’s best performing regimen could be improved by objective measurements.

Additionally, the cohort’s demographics and clinical characteristics are generally statistically indistinguishable from the MDS-UPDRS-III and PKG clustering methods. Only subjects with the longest disease duration were grouped into a separate cluster. This group required greater dosages and more frequent administration of dopaminergics for symptom control—a treatment strategy aligned with current clinical practice. Furthermore, using demographic information along with PKG time-series data yielded a classification model that enabled the random forest classifier to predict the cluster allocation of patients with high accuracy. A classification algorithm, such as one utilizing PKG measurements, could be used to streamline medication regimen optimization by providing a clinician with an estimate of a patient’s optimal regimen prior to clinical assessments. This would allow for a more complete view of the patient’s symptoms and medication response as the patient is continuously monitored throughout their daily lives. It is worth noting that using demographic information, MDS-UPDRS-III scores, and PKG time-series data resulted in the best performance and an incremental improvement (~3%) over the model that only used demographic information and PKG time-series data. However, models that do not include MDS-UPDRS-III scores can be used in remote settings where physicians may not have direct access to patients. Hence, the restrictions and considerations around access can select the right predictive model as part of a flexible decision support tool.

While the prognostication of disease progression is evident in clinical subtyping [26,33,34], the implications on treatment have yet to be established. Similar to the phenotypic variability of PD, the treatment approaches are also heterogeneous. Therefore, a continuous assessment of treatment response not only offers the possibility of more robust medication titrations, but the ability to cluster these sensor-based responses may help potentiate the impact of the emerging clinical phenotypes. This proof-of-concept study establishes that rich information can be extracted from time-series data collected from wearable sensors, such as the PKG—that measures both motor function and medication responses—and incorporated into ML algorithms to build predictive models capable of expanding the clinical treatment platform. 

### Limitations

The small patient sample is a limitation of this work. Due to the size of the examined patient cohort, a fully representative cluster of medication regimens was not likely achieved. This may be further biased, due to the underrepresentation of rare subtypes. By incorporating additional medication regimens in the future would provide a more comprehensive clustering scheme and would improve the optimized regimens estimates. 

Additionally, several subjects’ motor control symptoms within the cohort were never successfully controlled as measured by the PKG’s summary dyskinesia and bradykinesia scores [32]. Such subjects, when further optimized clinically, may be placed within a different cluster—altering the demographic and clinical information associated with that cluster. A more representative patient cohort followed longitudinally stands to enhance the clustering and possibly reveal other inherent treatment clusters. 

It should also be noted that the patients’ treatment regimens were optimized by clinicians considering both MDS-UPDRS-III scores and PKG’s summary dyskinesia and bradykinesia scores. This could further introduce bias into the classification algorithm as PKG measurements may perform better at predicting patients, in which physicians heavily utilized the PKG (namely those with motor fluctuations and dyskinesia) to determine their optimal regimens. Leave-one-out cross-validation was used in the analysis as each patient predicted by the random classifier is effectively a completely new patient—thus minimizing this potential source of bias. 

Finally, nonmotor symptoms were not directly considered in the determination of the best regimen. Therefore, regimen estimates that utilize sensors, such as the PKG, which primarily measures changes in motor symptoms, will need to be holistically considered by a physician. 

## 5. Conclusions

Clustering patients in a treatment-related manner has the potential to determine inherent regimen similarities within clinically relevant clusters. These inherent regimens are not necessarily specific to demographic groups. The identification of such regimen similarities could guide strategic treatment planning for a data-driven approach to refine clinical management, namely, by providing patients with wearable sensors to monitor their symptoms and medication responses. This approach can provide clinicians with estimated regimens based on the patients’ demographic information, clinical assessments, and wearable sensor measurements. The most accurate regimen estimations are achieved using a combination of clinical assessments and wearable sensors. This potentially expands the current approach for optimizing drug regimens in the clinic and remotely.

## Figures and Tables

**Table 1:** Patient demographics and clinical characteristics.

	Mean ± SD
Gender (female/male)	9/17
Age in years	71.19 ± 9.70
Age at diagnosis (years)	65.77 ± 10.37
Visit 1 MDS-UPDRS-III	28.89 ± 14.07
Visit 2 MDS-UPDRS-III	24.12 ± 13.50
Visit 1 H&Y stage	1.77 ± 0.71
Visit 2 H&Y stage	1.85 ± 0.78
Visit 1 levodopa equivalent dose (mg)	498.94 ± 309.88
Visit 2 levodopa equivalent dose (mg)	637.40 ± 322.37
Time between clinical visits (days)	65.62 ± 26.46

**Table 2:** Demographic and clinical information for clusters depicted in Figure 3a–d.

	Cluster A	Cluster B	Cluster C	Cluster D
Visit 2 MDS-UPDRS-III & PKG Scores				
Study age (years)	74.31 ± 9.99	67.96 ± 9.70	68.15 ± 3.96	68.29 ± 5.68
Age at diagnosis (years)	69.69 ± 9.22	65.41 ± 9.08	60.48 ± 10.32	54.95 ± 4.15 ^†^
Years of PD	4.62 ± 3.54	2.55 ± 1.78	7.67 ± 6.65	13.33 ± 2.05 ^†^
Gender (female/male))	5/8	2/5	1/2	1/2
Number of participants	13	7	3	3
Best MDS-UPDRS-III				
Study age (years)	75.62 ± 7.84	65.59 ± 10.86	63.54 ± 0.00	67.40 ± 3.74 ^††^
Age at diagnosis (years)	70.26 ± 7.67	62.90 ± 10.35	46.54 ± 0.00	59.90 ± 8.17
Years of PD	5.36 ± 4.55	2.69 ± 1.77	17.00 ± 0.00	7.50 ± 4.61
Gender (female/male)	5/9	2/5	0/1	2/4
Number of participants	14	7	1	4
Best PKG Scores				
Study age (years)	72.92 ± 10.21	67.66 ± 7.54	63.54 ± 0.00	70.93 ± 5.24
Age at diagnosis (years)	68.69 ± 9.47	63.49 ± 8.78	46.54 ± 0.00	57.43 ± 2.74
Years of PD	4.23 ± 3.31	4.17 ± 4.14	17.00 ± 0.00	13.50 ± 2.50 ^†^
Gender (female/male)	6/11	3/3	0/1	0/2
Number of participants	17	6	1	2

^†^ Pairwise *p*-value < 0.05 when compared with both Cluster A and B. ^††^ Pairwise *p*-value < 0.05 when compared with only Cluster A. Unless denoted by ^†^ or ^††^, each pairwise cluster comparison yields no statistical difference (*p*-value > 0.05).

**Table 3:** Dosage and medication types for clusters depicted in Figure 3c,d.

	Cluster A	Cluster B	Cluster C	Cluster D
Best MDS-UPDRS-III				
LEDD	387 ± 151	643 ± 127	1380 ± 0	1157 ± 183
Carbidopa/levodopa IR	279 ± 159	629 ± 150	1050 ± 0	925 ± 299
Carbidopa/levodopa CR	21 ± 80	--	200 ± 0	300 ± 476
Ropinirole	--	1 ± 4	4 ± 0	--
Selegiline	--	--	10 ± 0	1 ± 1
Rasagiline	0.2 ± 0.4	--	--	--
Rytary	194 ± 547	--	--	--
Best PKG Scores				
LEDD	381 ± 105	942 ± 233	1380 ± 0	1131 ± 206
Carbidopa/levodopa IR	335 ± 147	917 ± 183	1050 ± 0	250 ± 354
Carbidopa/levodopa CR	18 ± 73	33 ± 82	200 ± 0	500 ± 707
Ropinirole	--	--	4 ± 0	--
Selegiline	0.6 ± 2.4	--	10 ± 0	1 ± 2
Rasagiline	0.1 ± 0.3	--	--	0.3 ± 0.4
Rytary	45 ± 184	--	--	1170 ± 1655

Medications are aggregate results for each cluster and do not represent a single subject’s regimen.

**Table 4:** Random forest classifier performance identifying subjects in clusters A and B, as stratified through k-means clustering under the best PKG score. Each classifier was trained with a combination of features: Demographic information, demographic information and MDS-UPDRS-III scores (Visit 1), demographic information and PKG time-series data (Visit 1), and demographic information, visit MDS-UPDRS-III scores (Visit 1), and PKG time-series data (Visit 1).

	Demographics Alone	Demographics and MDS-UPDRS-III	Demographics and PKG	Demographics, MDS-UPDRS-III and PKG
Sensitivity	61.3 ± 1.0%	65.2 ± 0.8%	84.5 ± 0.7%	86.5 ± 0.5%
Specificity	62.3 ± 1.5%	66.0 ± 0.7%	81.7 ± 2.2%	87.7 ± 1.6%
Accuracy	61.6 ± 0.8%	65.4 ± 0.6%	83.8 ± 0.7%	86.9 ± 0.6%
PPV	82.2 ± 0.6%	84.4 ± 0.3%	93.1 ± 0.8%	95.3 ± 0.6%
F1 Score	70.1 ± 0.8%	73.5 ± 0.6%	88.5 ± 0.5%	90.7 ± 0.4%
AUC	0.618 ± 0.008	0.656 ± 0.005	0.831 ± 0.011	0.871 ± 0.008

## Data Availability

Restrictions apply to the availability of these data. Data was obtained from Nahab, F. [23] and are available from the corresponding author with the permission of F.B.N.

## Appendix A

A total of 1309 features were extracted and engineered from dyskinesia and bradykinesia time-series collected by PKG sensors using TSFresh [30]. Demographic information (patient’s age at visit 1, age at diagnosis, number of years experiencing PD symptoms, and gender) was also provided as features. The top ten most important features were included in the random forest analysis based on preliminary experiments. These parameters are presented in Table A1.

**Table A1:** Features included in the random forest analysis based on preliminary experiments.

Calculated Features	Description
**Dyskinesia**	
Dyskinesia ar coefficient k 10 coeff 2	Unconditional maximum likelihood of an autoregressive AR(k) process—coeff 2—k10
Dyskinesia fft coefficient coeff 39 attr “angle”	One-dimensional discrete fast Fourier transform—coeff 39—angle
Dyskinesia spkt welch density coeff 5	Cross power spectral density—coeff 5
**Bradykinesia**	
Bradykinesia agg autocorrelation f agg “mean” maxlag 40	Aggregation function fagg—mean maxlag—40
Bradykinesia agg linear trend f agg “mean” chunk len 50 att “rvalue”	Linear least-squares regression aggregated over chunks versus the sequence from 0 up to the number of chunks minus one—mean—chunk length 50—attribute rvalue
bradykinesia agg linear trend f agg “min” chunk len 50 attr “slope”	Linear least-squares regression aggregated over chunks versus the sequence from 0 up to the number of chunks minus one—mean—chunk length 50—attribute slope
Bradykinesia fft coefficient coeff 23 attr “abs”	One-dimensional discrete fast Fourier transform—coeff 23—abs
Bradykinesia fft coefficient coeff 94 attr “angle”	One-dimensional discrete fast Fourier transform—coeff 94—angle
Bradykinesia fft coefficient coeff 76 attr “imag”	One-dimensional discrete fast Fourier transform—coeff 76—imag
Bradykinesia index mass quantile q 0.9	Relative index i where q% of the mass of the time series x lie left of I-quantile 0.9

The Within Cluster Sum of Squares (WCSS) measurement is presented in Figure A1. The WCSS measurement seeks to minimize the within-cluster variance applied in k-means clusters for the number of cluster selections.
